# Pseudoaneurysm as an unusual complication in bone lengthening

**DOI:** 10.1016/j.jpra.2024.05.009

**Published:** 2024-05-31

**Authors:** Javier Martínez Ros, José Molina González, César Salcedo Cánovas, Dolores Abellán Rivero, Alicia Hernández Torres, Rubén Taboada Martín, Clemente Fernández Pascual, María Carrillo García, Miguel Martínez Ros, José Pablo Puertas García-Sandoval

**Affiliations:** a Unidad de Patología Séptica y Reconstructiva Osteoarticular, Servicio de Cirugía Ortopédica y Traumatología, Hospital Clínico Universitario Virgen de la Arrixaca Murcia, Spain; b Servicio de Radiodiagnóstico, Hospital Clínico Universitario Virgen de la Arrixaca Murcia, Spain; c Servicio de Medicina Interna y Enfermedades Infecciosas, Hospital Clínico Universitario Virgen de la Arrixaca Murcia, Spain; d Servde Cirugía Cirugía Cardiovascular, Hospital Clínico Universitario Virgen de la Arrixaca Murcia, Spain; e Servicio de Cirugía Plástica y Quemados, Hospital Clínico Universitario Virgen de la Arrixaca Murcia, Spain; f Servicio de Cirugía Ortopédica y Traumatología, Hospital Clínico Universitario Virgen de la Arrixaca Murcia, Spain

**Keywords:** Bone lengthening, Pseudoaneurysm, External fixation, Truelok, Infection, Vascular lesion

## Abstract

One of the risks of distraction osteogenesis-based techniques is the development of vascular complications, such as pseudoaneurysms associated with the osteotomies performed or the fixation elements of the external fixator used in the procedure.

Pseudoaneurysm are formed when the tunica adventitia of the artery is injured, resulting in a gradual and persistent blood extravasation into the surrounding tissues that is encapsulated and connected to the arterial lumen.

This report describes a rare case of a late-presentation pseudoaneurysm in the anterior tibial artery resulting from a tibial lengthening procedure aimed at addressing a leg length discrepancy in a 57-year-old female with severe peripheral neuropathy resulting from long-standing poorly controlled diabetes mellitus. We describe the diagnostic process, the treatment options and confirm how the shape of the bony callus can be a reliable indicator of this pathology, as has already been described in the literature.

## Introduction

Although distraction osteogenesis-based techniques are the standard of care for the management of septic bone defects, they are not exempt from risk.^1^ One of such risks is the development of vascular complications associated with the osteotomies performed and the eternal fixators applied which, although uncommon,^2^ have been well documented.^3^, ^4^, ^5^

Pseudoaneurysms occur as a result of a physical or biological injury to the tunica adventitia of the artery, which results in a gradual and persistent extravasation of blood into the surrounding tissues. After fibrous encapsulation and liquefaction of the center of the hematoma, the tissues form a mass that is connected to the arterial lumen.^5^ Their nature depends on their location, the size of the lesion and the surrounding structures. Although they can occur acutely as life-threatening hematomas,^6^ they are more likely to appear in the form of a painful pulsating inflammation.^2^^,^^5^

This report describes a pseudoaneurysm in the anterior tibial artery detected at four months from initiation of a tibial lengthening procedure. The case follows the STROBE guidelines. The patient gave her explicit informed consent to its publication.

## Case report

We present the case of a 57-year-old female with severe peripheral neuropathy resulting from diabetes mellitus. In 2017 she underwent a left tibiotalar arthrodesis, but aseptic loosening of the plate used led to its a reoperation with a retrograde nail and bone grafting. Evolution was unfavorable, with development of an infection that required multiple debridement surgeries, eventually leading to the removal of the fixation hardware with positive cultures for *Candida parapsilosis* and *Enterococcus faecium*. At that time the patient was referred to the Virgen de la Arrixaca Hospital (Table 1).

**Table 1:** Timeline of events.

2017	Tibiotalar arthrodesis with a specific plate
	Aseptic loosening the plate
	Ankle arthrodesis with an intramedullary nail and bone grafting
	Infection of the arthrodesis
	Several debridement surgeries
	Failure (and removal) of the fixation hardware. Diagnosis: septic tibiotalar nonunion
February 2021	Referral to the septic and reconstructive surgery unit of the Virgen de la Arrixaca Hospital
February 2021	Resection of the nonunion site and stabilization with a circular external fixator
April 2021	Proximal osteotomy in preparation for limb lengthening
August 2021	Diagnosis of pseudoaneurysm and surgical treatment
December 2021	Removal of the external fixator
October 2023	Final follow-up visit

Infection was accompanied by a 2 cm leg-length discrepancy, valgus deformity of the ankle, severe involvement of lateral soft tissues and a medial plantar ulcer. A decision was made to carry out an extensive bone and soft tissue debridement, with acute compression of the nonunion site and stabilization of the limb with a circular fixation system (TrueLok, Orthofix Srl, Italy). The surgery was carried out through a medial approach using skin Z-plasties. The nonunion site was resected in a 4-cm block, with soft tissue debridement, recanalization of the medullary cavity and bone refreshment. Despite the acute shortening of the limb, the use of Z-plasties made wound closure possible. A compressive tibiotalar arthrodesis was performed using the external fixator, whose proximal portion was left ready for a subsequent lengthening procedure aimed at compensating for the leg-length discrepancy. Intraoperative cultures were positive for *Candida parapsilosis*, prompting the implementation of a tailored treatment regimen by the hospital's infectious diseases unit.

**Figure 1:**
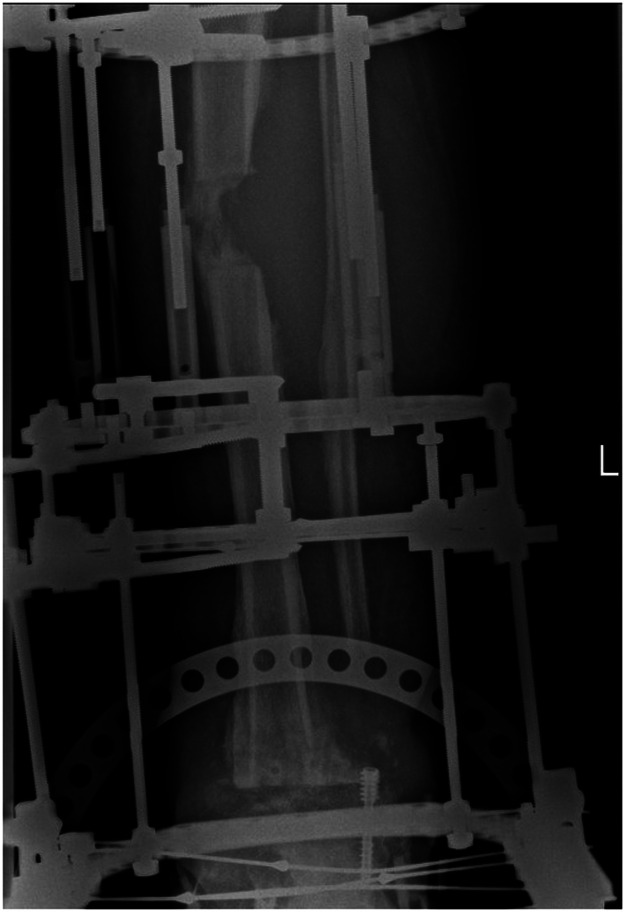
Radiographic image showing the deformity in the bone regenerate caused by the pressure exerted by the pseudoaneurysm.

**Figure 2:**
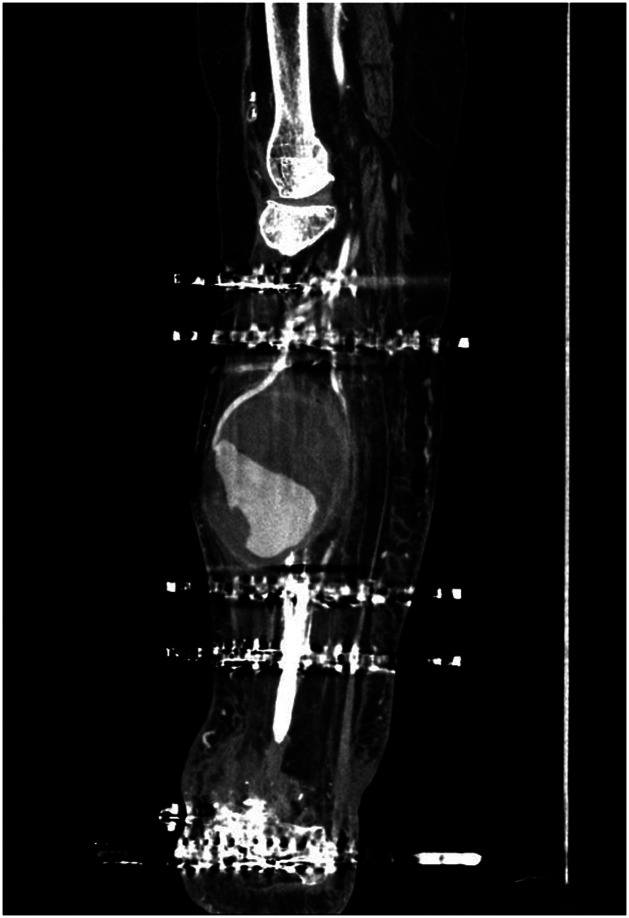
Sagittal computed tomography angiography-based MPR reconstruction showing a partially thrombosed pseudoaneurysm (hypodense upper portion) with a fine neck communicating it to the anterior tibial artery.

The patient evolved satisfactorily. At eight weeks post-op she underwent a proximal osteotomy and bone lengthening was initiated. The treatment proceeded uneventfully until completion the planned 5 cm increase in length during the lengthening phase. Four months after the osteotomy, the patient reported a suddenly growing mass in the middle third of the leg. A physical examination revealed a neoplasm of elastic consistency that was pulsatile on palpation. An ultrasonographic and CTA analysis confirmed the presence of a large pseudoaneurysm (9.9 cm x 10 cm) arising from the anterior tibial artery presenting with moderate inflammation in its walls and in the surrounding adipose tissue (Figure 1, Figure 2). In agreement with vascular surgery, emergency surgery was performed to address the iatrogenic pseudoaneurysm identified at the osteotomy site. Ligation of the anterior tibial artery was performed and the resulting dead space was obliterated using vancomycin-loaded calcium sulphate beads (Stimulan, Biocomposites, UK).

**Figure 3:**
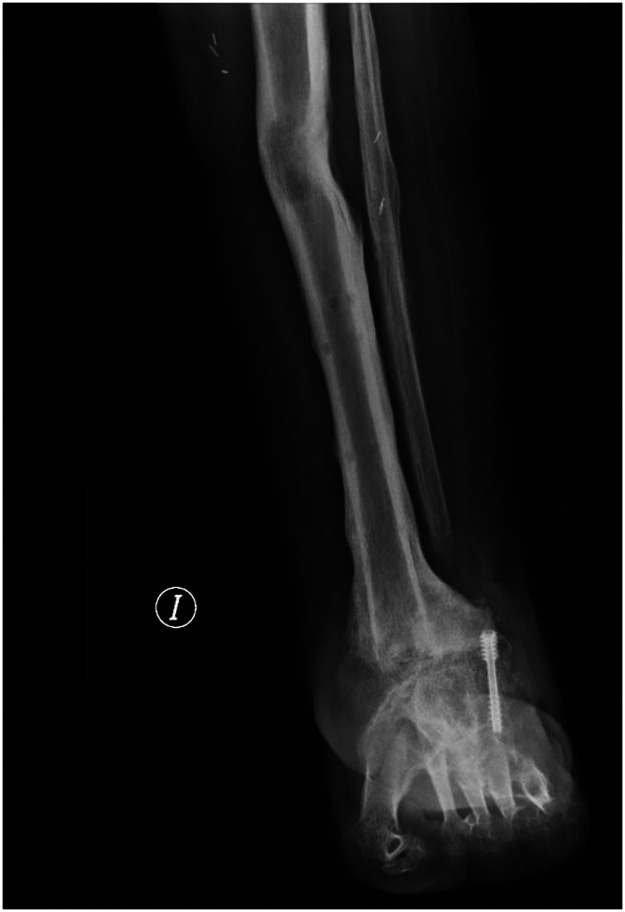
Patient's final radiographic status, displaying a residual deformity resulting from the pressure exerted by the pseudoaneurysm.

Ten months after the start of treatment (external fixation index: 2 months/cm) and eight months from the osteotomy (bone healing index: 1.6 months/cm) a decision was made to remove the external fixator given that both the tibiotalar arthrodesis and the maturation of the regenerate had been successful (Figure 3).

Twenty-two months into follow-up, the patient is infection-free and able to independently perform her daily activities.

## Discussion

Vascular injuries are a severe complication associated with external fixation treatments. They are typically caused by the tips of external fixator pins, a careless drilling technique, the presence of bone fragments, the retraction of the surrounding tissue or the performance of osteotomies.^7^ Fortunately, the incidence of such injuries is relatively low, with reported rates ranging between 1.5 % and 2.5 %.^2^ Occurrence of pseudoaneurysms in these situations is rare.^2^^,^^4^, ^5^, ^6^ In fact, pseudoaneurysms are not considered a complication resulting from bone lengthening procedures since they usually occur during surgery, due to injury caused by the drills or the external fixator pins.

Vascular injuries are consequently detected immediately after surgery. Swelling of the leg, bleeding from the wound and/or decreased hemoglobin levels are important warning signs.^7^ However, detection of slow-growing pseudoaneurysms is typically incidental.^6^ Similarly to other authors, in our case the pseudoaneurysm was identified several months postoperatively as an elastic pulsating mass.^2^^,^^8^^,^^9^ The literature does contain, however, reports of non-pulsating masses^7^ and even of isolated bleeding episodes.^5^

The imaging techniques proposed in the literature for the diagnosis of pseudoaneurysms include simple^6^^,^^8^ and Doppler^2^^,^^5^ ultrasonography, simple angiography,^6^, ^7^, ^8^ computed tomography angiography (CTA)^4^^,^^5^ and magnetic resonance imaging.^5^ We used ultrasonography to establish a preliminary diagnosis, which was subsequently confirmed by means of CTA and contrast-enhanced angiography, a more accurate and less invasive technique. These tests revealed that the neck of the pseudoaneurysm was located at the level of the osteotomy, suggesting iatrogenic injury during surgery, probably while drilling for the DeBastiani technique. Several osteotomy techniques have been described for distraction osteogenesis. The most common ones are the Ilizarov technique, DeBastiani's percutaneous multiple drill hole technique and the Afghan technique using a Gigli saw. The literature seems to favor the use of techniques that minimize local trauma, reduce thermal necrosis, and do not restrict blood supply to the area. At any event, although there are no studies comparing the incidence of vascular injuries with the different options, Paley et al. found that the biggest risk associated with the Afghan technique was, precisely, iatrogenic vascular damage. It must be said, however, that the incidence of these lesions with the different techniques is extremely low.

An intriguing finding in the radiographic analysis was a concave deformation of the bone regenerate (Figure 1), which was attributed to the mechanical pressure exerted by the pseudoaneurysm. This kind of deformity was reported by Fagg et al.,^6^ who described it as *asymmetrical scalloping*, suggesting that its presence on one side of the regenerate could be indicative of a pseudoaneurysm and should lead to a more in-depth analysis. Although our case would seem to support this theory, the regenerate in Fagg et al. regained its normal shape following embolization of the pseudoaneurysm while our patient retained a slight deformity probably because the regenerate had already reached an advanced stage during the healing process.

As regards the treatment, watchful waiting may be an option for smaller pseudoaneurysms as they often resolve spontaneously.^2^ Nevertheless, the size of our lesion precluded that alternative. Untreated pseudoaneurysms may result in major complications such as rupture and hemorrhage, loss of blood supply, migration of emboli, infection and local pressure on neighboring structures.^6^ The treatments described in the literature include selective embolization,^5^, ^6^, ^7^, ^8^, ^9^ ultrasound-guided compression,^2^^,^^5^^,^^8^ endovascular repair with percutaneous thrombin injections,^2^^,^^5^ resection and end-to-end anastomosis with a venous graft,^9^ lateral suturing^9^ and endoaneurysmorrhaphy.^9^ Treatment selection should depend on the size and location of the pseudoaneurysm,^9^ with several authors arguing that the treatment of choice in aneurysms of one of the three arteries of the leg must be selective embolization, provided that the remining arteries are able to provide sufficient collateral flow.^5^^,^^6^^,^^8^

Intraoperatively, our suspicion of iatrogenic damage was confirmed. Ligation of the anterior tibial artery was successful and the resulting dead space was prophylactically obliterated with antibiotic-impregnated calcium sulphate beads. Two years after removal of the fixator, the patient remains infection-free and functionally independent.

## Conclusion

External fixation is a valuable tool, but it is not without risks. Although vascular complications are rare, their potential severity underscores the need for meticulous surgical technique and vigilant monitoring of clinical signs.

## Funding

None.

## Ethical approval

Informed consent given by the patient for publication of the report.

## Declaration of competing interest

None declared.
